# Progressive Systemic Sclerosis With Negative Antinuclear Antibodies and Absence of Raynaud’s Phenomenon: A Case Report and Literature Review

**DOI:** 10.7759/cureus.35663

**Published:** 2023-03-01

**Authors:** Anna C Falls, Catherine Wrigley, Surabhi A Khanna

**Affiliations:** 1 Division of Immunology, Allergy and Rheumatology, University of Cincinnati Medical Center, Cincinnati, USA; 2 Department of Rheumatology, Christie Clinic, Champaign, USA; 3 Division of Immunology, Allergy and Rheumatology, University of Cincinnati College of Medicine, Cincinnati, USA

**Keywords:** raynaud’s phenomenon, scleroderma renal crisis, interstitial lung disease, anti-nuclear antibody, systemic sclerosis

## Abstract

Systemic sclerosis (SSc) is typically characterized by positive antinuclear antibodies (ANA) and Raynaud’s phenomenon (RP). We present the case of a male patient with progressive diffuse skin tightening, interstitial lung disease (ILD), pericardial tamponade, renal failure, and gastrointestinal dysmotility who was diagnosed with severe, rapidly progressive SSc despite negative ANA, absent RP, and a negative malignancy workup. The patient’s clinical course was complicated by scleroderma renal crisis (SRC) requiring dialysis and eventual kidney transplantation. He also had severe gastrointestinal dysmotility requiring gastrostomy tube placement and total parenteral nutrition. Multiple agents were required for treatment, including mycophenolate mofetil (MMF) and rituximab. The patient eventually had improvement in his skin fibrosis and has been doing well in follow-up after kidney transplantation. Treatment of SSc can be challenging given the heterogeneity of the disease, and recognition of this subset of SSc patients is needed to help prevent early mortality among them.

## Introduction

Systemic sclerosis (SSc) is characterized by three pathological hallmarks according to the 2013 American College of Rheumatology (ACR)/European Alliance of Associations for Rheumatology (EULAR) classification criteria. These include excessive collagen deposition leading to fibrosis of the skin and internal organs, vasculopathy, and immune dysregulation [1]. Up to 95% of SSc patients are antinuclear antibody (ANA)-positive, and 99.8% have been reported to have either Raynaud’s phenomenon (RP) or a positive ANA [2-5]. The disease can, however, exist in the absence of positive ANA titer and/or RP. The clinical phenotype of ANA-negative SSc patients tends to differ from those with positive ANA titers [5-6]. ANA-negative SSc patients tend to have more fibrotic changes such as skin thickening or gastrointestinal involvement [5-6]. Vasculopathic manifestations such as RP are seen more often in ANA-positive patients [5-6]. We report the case of a patient with progressive diffuse skin tightening, interstitial lung disease (ILD), pericardial tamponade, renal failure, and gastrointestinal dysmotility who was diagnosed with severe, rapidly progressive SSc despite negative ANA and absence of RP.

## Case presentation

A 52-year-old Caucasian male with a past medical history of hypertension, obstructive sleep apnea, and hyperlipidemia presented to the rheumatology clinic in June 2016 for evaluation of cough, shortness of breath, generalized swelling, and a four-month history of skin puffiness and thickening. He had a negative malignancy evaluation including negative positron emission tomography (PET) scan. The patient denied any history of RP. He had a recent admission for possible pneumonia, but his shortness of breath did not improve significantly with antibiotics. Physical exam revealed normal vital signs, and skin thickening of fingers, hands, forearms, feet, and legs with a modified Rodnan skin score (mRSS) of 16 out of 51 (moderate to severe cutaneous involvement). Skin thickness progression rate (defined as mRSS at the first visit divided by the duration of skin thickening, in years) was 64 [7]. Manual muscle testing showed 5/5 strength in the upper and lower extremities. Bibasilar lung crackles and bilateral pitting pedal edema were noted. No tendon or bursal friction rubs were palpated, and nailfold capillaries were normal. Initial laboratory testing is summarized in Table 1.

**Table 1:** Initial laboratory evaluation

Laboratory test	Value	Reference range
Creatine kinase (CK)	998 U/L	30–223 U/L
Aldolase	18.4 U/L	3.3–10.3 U/L
Aspartate aminotransferase (AST)	53 U/L	13–39 U/L
Alanine aminotransferase (ALT)	45 U/L	7–52 U/L
Urinalysis	30 mg/dL protein and negative blood; negative protein on repeat testing	Negative
SSc antibodies (ANA by immunofluorescence, anti-SCL-70, RNA polymerase 3, anti-centromere, Th/To, U1RNP, fibrillarin/U3RNP, anti-PM/SCL, SSA and SSB)	Negative	Negative
Myositis antibodies (anti-Jo 1, Mi-2, TIF1 gamma, MDA-5, NXP-2, Ku, SRP, PL-7, PL-12, EJ, OJ, U2 snRNP, SSA-52, Scl-75, and Scl-100)	Negative	Negative

**Figure 1:**
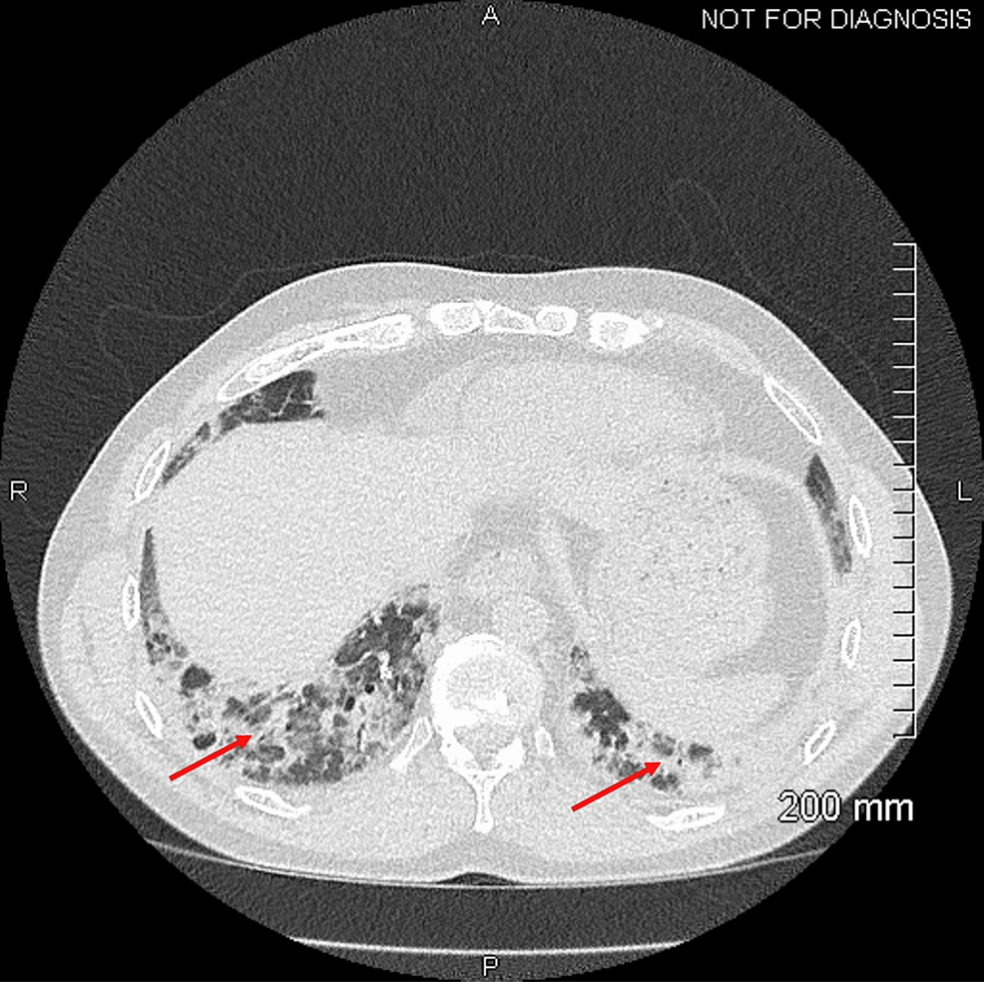
Non-contrast CT chest Axial view with bibasilar ground-glass opacities as indicated by arrows CT: computed tomography

CT chest showed bibasilar ground-glass opacities, lingular consolidation, and esophageal dilatation (Figure 1). Pulmonary function tests were initially limited due to patient effort, but repeat tests showed severe restriction with forced vital capacity (FVC) of 47% and diffusing capacity for carbon monoxide (DLCO) of 72%. Transthoracic echocardiogram was normal. EMG of the left upper and lower extremities showed a normal recruitment pattern with no positive waves or fibrillation potentials, inconsistent with inflammatory myopathy. The height of the motor units was on the small side of normal. Based on this information, the patient was diagnosed with SSc and SSc-related myopathy. He was started on a short course of prednisone 30 mg twice daily along with mycophenolate mofetil (MMF).

**Figure 2:**
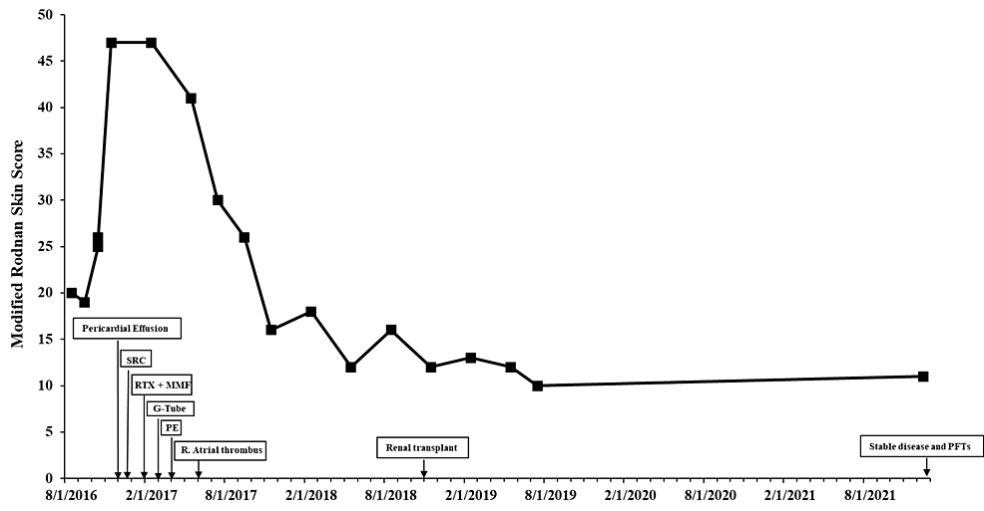
Modified Rodnan skin score and significant disease events over time

**Figure 3:**
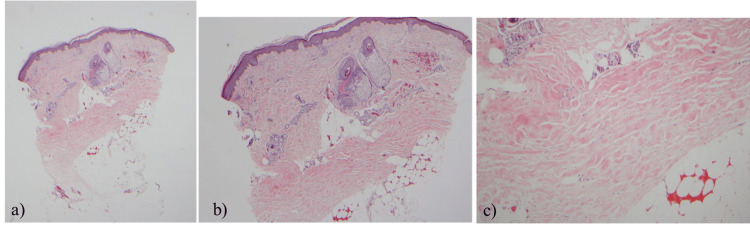
Left-arm skin biopsy a) 2x magnification - punch specimen with slight square biopsy sign and flattening of the dermal-fat junction. b) 4x magnification - sclerosis in the deep dermis in the form of the hypocellular collagen bundles with loss of ground substance between bundles. No inflammation is present. c) 10x magnification - sclerosis in the deep dermis in the form of the hypocellular collagen bundles with loss of ground substance between bundles. No inflammation is present

Over the next four months, the patient's mRSS continued to progress from 16 to 25 (Figure 2). Skin biopsies of bilateral forearms showed non-specific findings of slight vascular hyperplasia (right) and possible deep dermal fibrosis and sclerosis (left, Figure 3). After the skin biopsy, the patient's disease kept worsening with progressive skin fibrosis (Figure 2). His course continued to worsen, and the patient was admitted to the hospital for pericardial effusion with tamponade physiology and underwent a pericardial window procedure. He was treated with oral cyclophosphamide in place of mycophenolate and prednisone 10 mg twice daily, which was later tapered down. The postoperative course was complicated by blurry vision, elevated blood pressure (systolic peak of 179 and diastolic peak of 112 mmHg), anemia with hemolysis, and thrombocytopenia. The patient was found to be in acute renal failure and was promptly started on captopril for scleroderma renal crisis (SRC). Unfortunately, his renal function continued to worsen, and the patient became dialysis-dependent. He also developed retinal ischemic changes. Rituximab 1000 mg IV x two doses was initiated in combination with MMF in February 2017. Over the next month, the patient’s gastrointestinal symptoms worsened with significant dysphagia and inability to tolerate oral intake, and he required gastrostomy tube placement and eventually total parenteral nutrition due to failure to thrive. In April 2017, the patient developed a pulmonary embolism and was started on anticoagulation, which had to be held due to a small intracranial hemorrhage. He was subsequently found to have a right atrial thrombus in June 2017 when anticoagulation was resumed. Hypercoagulable workup and antiphospholipid labs were negative. His skin score improved steadily to 16 in November 2017 (Figure 2). He underwent renal transplantation in November 2018 and has been doing well overall. He is now on MMF and tacrolimus for transplant maintenance, which he is tolerating and is adherent to based on serologic monitoring. In December 2021, his skin score was 10, and lung disease remains stable with FVC at 50% and DLCO at 55%. He is tolerating the oral diet well, and his weight remains stable. Repeat serologies and complete scleroderma panel remain negative.

## Discussion

While positive ANA and RP are often thought to be diagnostic hallmarks of SSc, this case illustrates that their absence does not exclude the diagnosis and other severe disease manifestations may still be present. Autoantibodies have been associated with distinct clinical features in SSc, but their pathogenic effect has not been well established [4,8]. ANA is not believed to play a significant role in pathogenesis but is an immunological marker of the disease [2]. Two multicenter cohort studies have evaluated ANA-negative SSc patients and predominantly found fibrosis and fewer vasculopathy features [5-6]. In the EULAR cohort, only 12 out of 5390 patients lacked both ANA and RP [5]. They trended towards shorter disease duration, higher Rodnan skin scores, and more diffuse disease [5]. Four of the seven patients for whom detailed information was available had malignancy [5]. In the Scleroderma Family Registry and DNA repository, 208 out of 3249 patients (6.4%) were ANA-negative [6]. ANA-negative patients were more commonly male, had higher DLCO, more gastrointestinal involvement, and fewer vasculopathy features compared to ANA-positive patients [6]. The ANA-negative group more commonly had diffuse disease but lower Rodnan skin scores overall, which is in contrast to our case [6].

Exhaustive cancer screening must be performed in patients with ANA-negative SSc due to the high prevalence of this condition as a paraneoplastic phenomenon [5]. More and more studies in the literature suggest a temporal clustering of cancer with scleroderma in patients with positive RNA polymerase III antibodies and in those with triple-negative antibodies (negative RNA polymerase III, centromere, and topoisomerase-1) [9]. Our case highlights that, albeit rare, those with ANA-negative systemic sclerosis do constitute a small subset of scleroderma patients. In a small study involving 37 patients, no evidence of malignancy was found in the five ANA-negative patients [10].

Recognition of this rare clinical presentation of SSC is critical as pericardial effusions, SRC, and pulmonary fibrosis can be seen with this. In the Scleroderma Family Registry and DNA repository, ANA-negative patients were more likely to develop SRC than patients with anti-topoisomerase or anti-centromere antibodies [6]. Our patient had risk factors for the development of SRC, including antecedent use of steroids (for pericardial tamponade), pericardial effusion, early disease (<4 years from diagnosis), and diffuse disease with rapidly progressive skin score [7,11]. SSc has a substantial burden of early mortality within five years of disease onset, although all-cause mortality was found to be similar between ANA-negative and ANA-positive patients, with ILD being the leading cause [6,12].

Treatment of scleroderma can be challenging given the heterogeneity of the disease and the lack of standardized treatment algorithms. Therapeutic options are often guided by the type and severity of organ manifestations. Combination therapy is often needed for comprehensive management, and multiple investigations of novel agents (including anti-fibrotic and biologic therapies) are underway in phase 3 and 4 clinical trials [13]. Our patient continued to have disease progression on MMF and oral cyclophosphamide monotherapies but notably started improving after combination therapy with rituximab and MMF.

## Conclusions

Overall, ANA-negative SSc patients may constitute a rare subset of this patient group with distinct presentation seen more commonly in males, with more fibrotic manifestations, such as diffuse skin tightening and malabsorption, and fewer vasculopathy manifestations, such as pulmonary hypertension, telangiectasias, RP, and digital ulcerations. The incidence of ILD, SRC, and overall survival are notably similar to that in ANA-positive patients; however, ANA-negative patients more commonly have SRC than those with anti-topoisomerase or anti-centromere antibodies.

Clinicians must be aware of ANA-negative, scleroderma-specific antibody-negative, and RP-negative systemic sclerosis. Cancer workup must be performed, but not all ANA-negative scleroderma cases represent a paraneoplastic phenomenon. Timely recognition of the disease and vigilance for SRC is important given the potential for severe organ involvement as well as early mortality.
